# Use of social service counseling by cancer patients: an analysis of quality assurance data of 6339 breast cancer patients from 13 certified centers in Germany treated between 2015 and 2017

**DOI:** 10.1186/s12885-021-08396-1

**Published:** 2021-06-05

**Authors:** Clara Breidenbach, Simone Wesselmann, Nora Tabea Sibert, Olaf Ortmann, Katrin Blankenburg, Cindy Stoklossa, Gerhard Gebauer, Marina dos Santos Guilherme, Christoph Lindner, Susanne Peschel, Friedemann Schad, Paul Strecker, Lorenz Rieger, Julia Ferencz, Sebastian Dieng, Christoph Kowalski

**Affiliations:** 1 German Cancer Society, Berlin, Germany; 2 Caritas-Krankenhaus St. Josef, Regensburg, Germany; 3 Deutsche Vereinigung für Soziale Arbeit im Gesundheitswesen e.V., Berlin, Germany; 4 Asklepios Klinik Barmbek, Hamburg, Germany; 5 St. Vincenz-Krankenhaus Limburg, Limburg, Germany; 6 Agaplesion Diakonieklinikum Hamburg, Hamburg, Germany; 7 St. Bernward Krankenhaus, Hildesheim, Germany; 8 Gemeinschaftskrankenhaus Havelhöhe, Berlin, Germany; 9 Helios Klinikum Erfurt, Erfurt, Germany; 10 Krankenhaus Landshut-Achdorf, Landshut, Germany; 11 OnkoZert GmbH, Neu-Ulm, Germany

**Keywords:** Psychosocial counseling, Social service counseling, Certification, German Cancer Society, Quality indicators

## Abstract

**Background:**

Integrated social care may help to mitigate social risk factors in order to achieve more equitable health outcomes. In cancer centers certified according to the criteria set out by the German Cancer Society, every patient must be given low-threshold access to qualified social workers at the center for in-house social service counseling (SSC). Previous analyses have demonstrated large variation in the utilization of these services across individual centers. Therefore, this research aims at investigating whether SSC utilization varies regarding breast cancer patient characteristics and center characteristics presenting a unique approach of using routine data.

**Methods:**

Multilevel modeling was performed using quality assurance data based on 6339 patients treated in 13 certified breast cancer centers in Germany in order to investigate whether SSC utilization varies with patient sex, age, and disease characteristics as well as over time and across centers.

**Results:**

In the sample, 80.3% of the patients used SSC. SSC use varies substantially between centers for the unadjusted model (ICC = 0.24). Use was statistically significantly (*P* < .001) more likely in women, patients with invasive (in comparison to tumor in situ/ductal carcinoma in situ) diseases (*P* < .001), patients with both breasts affected (*P = .*03), patients who received a surgery (*P* < .001), patients who were diagnosed in 2015 or 2017 compared to 2016 (*P* < .001) and patients older than 84 years as compared to patients between 55 and 64 years old (*P* = .002).

**Conclusion:**

The analysis approach allows a unique insight into the reality of cancer care. Sociodemographic and disease-related patient characteristics were identified to explain SSC use to some extent.

**Supplementary Information:**

The online version contains supplementary material available at 10.1186/s12885-021-08396-1.

## Background

Cancer patients are not only burdened by physical consequences of the disease and its treatment, but also by psychosocial hardships as a result of the malignant disease. Besides the psychological processes of adapting to the disease, and the increased likelihood of developing a mental disorder, many patients experience social or financial strain and are troubled by the paperwork that is necessary to receive support [1–6]. Including health care workers providing social service counseling (SSC) into the professional team might help mitigating this psychosocial strain and giving practical assistance. Despite the well-described psychosocial burden associated with a cancer diagnosis, access to SSC is limited in many health care systems. This may be due to costs and reimbursement structures, lack of qualified staff, or lack of pathways that help patients find adequate providers [7]. Often, access to services only takes place after a substantial delay when timely interventions are needed [8]. The National Academy of Sciences of the USA only recently issued a report that urges for the integration of “social care into the delivery of health care” [9]. In that report, the authors argue that integrated social care may help to mitigate social risk factors to achieve more equitable health outcomes. A recent survey implies that higher SSC frequencies in breast cancer centers might relate to decreased information needs regarding financial problems and problems with health insurance [10].

To meet patients’ needs for timely counseling, requirements for the certification of cancer centers by the German Cancer Society demand that every patient must be given low-threshold, fee-free access to qualified social workers as part of the interprofessional team at the center. Counseling goals and content have been described elsewhere in detail [11–14] and include, among others, the identification of social, economic and psychological crises, the initiation of medical rehabilitation measures as well as advice on financial questions and social law. In addition to SSC which mainly focuses on practical help for the patients, like paperwork required to initiate medical rehabilitation, all patients have the right to see a psycho-oncologist on-site for immediate psychological counseling. These services and its utilization have been described previously for prostate cancer centers [15]. SSC counseling goals and contents are consistent across all cancer center types (e. g. prostate cancer center or breast cancer center). Certified centers are mandated to document the structures, processes, and outcomes of care [16], including the percentage of patients that receive SSC. In the past, analyses of this documentation have described differences in counseling frequencies between centers, different types of cancer, and over time. For instance, noticeable differences between the types of centers were observed, with the highest counseling frequencies in breast cancer centers [11, 12]. Due to the aggregated nature of the data, these analyses were limited and could not identify individual patient characteristics associated with the use of SSC. Investigating such associations may help identify groups with counseling needs more precisely and describe patterns of psychosocial counseling more rigorously. This is important since knowledge on patterns of psychosocial counseling in oncology is still limited [1, 17], while, at the same time, awareness of psychological, social, and financial issues associated with the disease is constantly growing [2] and social workers may help to address these needs in a timely manner.

This analysis presents a unique approach for giving insight into the reality of cancer care. It uses more granular quality assurance data from cancer centers to address the shortcomings of earlier reports. The objective is to identify associations of cancer patients’ utilization of SSC with patient sex, age, and disease characteristics. In addition, we investigated changes over time and across centers and center characteristics. The analysis is exemplary, based on quality assurance data collected in breast cancer centers in 2015–2017.

## Methods

### Data collection

Data used in this analysis are documented in certified breast cancer centers as part of the annual audit within the certification process. Documentation of data is an integral part of quality assurance; hospitals are obliged to conduct quality assurance according to § 135a of the German Social Code V (SGB V)^1^ and no patient consent is necessary. Centers document the information on a patient-by-patient basis in locally available software (a tumor documentation system). Currently, more than 20 different tumor documentation systems are in use in breast cancer centers, which differ in terms of user interface, field definition and algorithms. Data were collected with the help of the software tool OncoBox Research. The OncoBox Research serves to standardize the documentation fields and make the documentation comparable. It also checks whether data in the tumor documentation system are plausible and complete and asks the user to correct the data if necessary. Data was transferred pseudonymized. The dataset from the OncoBox Research contains information about the breast cancer center, technical information about the tumor documentation system, patient information about the diagnosis/histology, therapy details, short-term clinical outcomes, follow-up data and processual information. The primary purpose of the data collected is clinical quality assurance. Besides age and sex, there is no sociodemographic information provided.

### Study sample

Only patients with newly diagnosed invasive breast cancer or tumor in situ (TIS)/ductal carcinoma in situ (DCIS) between 2015 and 2017 of centers sending data for all three years were included in the analysis. Analysis was limited to so called “primary cases”. A primary case in this analysis was defined as a patient, i.e., not as a “stay” or a “procedure”, with first diagnosis of invasive breast cancer or TIS/DCIS who receives the majority of treatment in the center, including therapy planning (interdisciplinary tumor conference) and conduct of therapy by the breast cancer center (main therapy). Cases were only counted for one center and count time was the date of initial diagnosis. Breast cancer in men and newly diagnosed metastatic patients were counted as primary cases, and patients with bilateral breast cancer were counted as one case in order to prevent statistical double-weighting of one patient.

### Measures

Dependent variable: Patients were counted as having received counseling if they received SSC by one of the centers on an inpatient or outpatient basis during any phase of their treatment in the center. Patients were counted as not having received counseling if they received no inpatient or outpatient SSC by a center. If there were two primary cases per patient (due to bilateral breast cancer) and if it was consistently documented that either SSC utilization took place or no SSC utilization took place in both cases, the second primary case per patient was excluded from this analysis. If SSC utilization was documented inconsistently for the two cases, the case with the positive SSC utilization documented was included, and the other case excluded from this analysis, as, in this circumstance, the patient received SSC at least once. If it was documented that one case did not receive SSC and the other case had a missing value for SSC utilization, the case with a missing value was counted because it was uncertain whether the patient received SSC eventually, and was therefore excluded from the analysis.

Independent variables for patient characteristics: Sex (coded as female and male); age (grouped as younger than 35, 35–44, 45–54, 55–64, 65–74, 75–84, and older than 84 year); prior cancer diagnosis (yes, no), T-staging (grouped as TIS/DCIS, invasive, TX); N-staging (N0, >N0, NX); M-staging (M0, M1); whether both breasts were affected (yes, no); therapy type (surgery with recommendation for chemotherapy, surgery without recommendation for chemotherapy, no surgery documented); and year of diagnosis (2015, 2016, 2017).

Independent variables for center characteristics: Municipality (< 20,000 population, 20,000–100,000 population, > 100,000 population); teaching status (none, academic, university); ownership (private, not-for-profit), number of primary cases (continuous); and years since first certification (continuous).

### Data analysis

Descriptive statistics were calculated for all variables. First, univariate logistic multilevel analyses were calculated in order to show independent correlations of each variable with the outcome. Then, a multilevel analysis was performed with breast cancer patients on the first level and certified breast cancer centers on the second level. In order to determine the intraclass correlation coefficient (ICC), a two-level random intercept hierarchical logistic model without predictors was calculated first (null model). After that, patient and center characteristics were added to the model in order to examine how they account for SSC utilization. Relevant variables for the final model were chosen in a theory-driven manner. In order to test for differences between surgery types and SSC utilization, therapy type variables were also regrouped alternatively according to surgery types (mastectomy, breast-conserving surgery, no surgery) and an additional analysis was performed (details available upon request). Missing cases in the independent categorical variables were included as additional categories in order to avoid case deletion. To consider the collapsing of two “cases” into one “patient” for patients with two affected breasts in the analysis, the model was adjusted for occurrence of cancer in both breasts. It was not differentiated between metachronous and synchronous cases because of an unclear distinction between the two terms. In order to estimate the multilevel models, one case was excluded due to missing values. All statistical analyses were carried out using STATA, version 15.1 (StataCorp LLC, College Station, Texas, USA).

## Results

Data from 13 breast cancer centers were included in the analysis, providing information on 6339 primary cases. 5092 (80.3%) patients in the sample used SSC, and 1247 (19.7%) patients did not. Utilization of SSC ranged from 27.9 to 95.2% between the centers (median: 83.3%). Table 1 presents frequencies for the categorical variables and (center) means and standard deviations for continuous variables. Results of the univariate logistic multilevel analyses are presented in supplementary material 1: Correlations show statistically significant associations for sex, age, prior cancer diagnosis, T-staging, N-staging, M-staging, therapy type and date of diagnosis (Tables 1.1–1.6, 1.8–1.9, supplementary material 1). No statistically significant associations were found for both breasts affected (Table 1.7, supplementary material 1) and the center characteristics municipality, teaching status, ownership, number of primary cases, years since first certification (Table 1.10–1.14, supplementary material 1).

**Table 1:** Descriptive results at patient level for all patients in the sample (*n* = 6339)

Variable	Response option	n (%) total	n (%) with SSC	n (%) without SSC
Patient characteristics				
Sex	Female	6292 (99.3)	5062 (99.4)	1230 (98.6)
	Male	47 (0.7)	30 (0.6)	17 (1.4)
Age	Younger than 35 years	87 (1.4)	74 (1.5)	13 (1.0)
	35–44 years	403 (6.4)	332 (6.5)	71 (5.7)
	45–54 years	1330 (21.0)	1102 (21.6)	228 (18.3)
	55–64 years	1477 (23.3)	1201 (23.6)	276 (22.1)
	65–74 years	1434 (22.6)	1183 (23.2)	251 (20.1)
	75–84 years	1292 (20.4)	1006 (19.8)	286 (22.9)
	Older than 84 years	316 (5.0)	194 (3.8)	122 (9.8)
Prior cancer diagnosis	Yes	170 (2.7)	118 (2.3)	52 (4.2)
	No	6169 (97.3)	4974 (97.7)	1195 (95.8)
T-staging	TIS/DCIS	581 (9.2)	411 (8.1)	170 (13.6)
	Invasive breast cancer	5736 (90.5)	4668 (91.7)	1068 (85.7)
	TX	22 (0.4)	13 (0.3)	9 (0.7)
N-staging	N0	3841 (60.6)	3140 (61.7)	701 (56.2)
	> N0	2071 (32.7)	1650 (32.4)	421 (33.8)
	NX	427 (6.7)	302 (5.9)	125 (10.0)
M-staging	M0	5950 (93.9)	4854 (95.3)	1096 (87.9)
	M1	389 (6.1)	238 (4.7)	151 (12.1)
Both breasts affected (metachronous and synchronous)	No	6046 (95.4)	4857 (95.4)	1190 (95.4)
	Yes	292 (4.6)	235 (4.6)	57 (4.6)
Therapy type	Surgery with recommendation for chemotherapy	2145 (33.8)	1853 (36.4)	292 (23.4)
	Surgery without recommendation for chemotherapy	3779 (59.6)	3097 (60.8)	682 (54.7)
	No surgery	415 (6.6)	142 (2.8)	273 (21.9)
Date of diagnosis	2015	2105 (33.2)	1686 (33.1)	419 (33.6)
	2016	2153 (34.0)	1611 (31.6)	542 (43.5)
	2017	2081 (32.8)	1795 (35.2)	286 (22.9)
Center characteristics				
Municipality	< 20,000 population	411 (6.5)	347 (6.8)	64 (5.1)
	20,000–100,000 population	4153 (65.5)	3208 (63.0)	945 (75.8)
	> 100,000 population	1775 (28.0)	1537 (30.2)	238 (19.1)
Teaching status	None	567 (8.9)	365 (7.2)	202 (16.2)
	Academic	5003 (78.9)	4083 (80.2)	920 (73.8)
	University	769 (12.1)	644 (12.7)	125 (10.0)
Ownership	Not-for-profit	6063 (95.6)	4859 (95.4)	1204 (96.6)
	Private	276 (4.4)	233 (4.6)	43 (3.4)
Number of primary cases	Continuous	6339 (100) Center Mean (SD): 142.5 (9.7)		
Years since first certfiication	Continuous	6339 (100) Center Mean (SD): 11.1 (5.9)		

*Note*: *TIS* tumor in situ, *DCIS* ductal carcinoma in situ, *SD* standard deviation

Table 2 presents the results of the final multilevel analysis. The null model, without predictors, revealed an ICC of 0.24. The final multilevel model demonstrated that men were less likely to use SSC compared to women (odds ratio (OR) 0.32; 95%-confidence interval (95 CI) 0.16–0.64). Age (OR 0.57; 95 CI 0.40–0.81) was significantly associated with SSC use, with patients older than 84 years being less likely to use SSC than patients aged 55–64 years. Patients with a TIS/DCIS were also less likely to utilize SSC than patients with invasive breast cancer (OR 0.47; 95 CI 0.28–0.49). When both breasts were affected (metachronous and synchronous), patients had higher odds to utilize SSC than patients with one breast affected (OR 1.48; 95 CI 1.03–2.11). Patients without surgery were less likely to utilize SSC, compared to patients with a surgery without recommendation for chemotherapy (OR 0.08; 95 CI 0.06–0.11). Patients diagnosed in 2015 (OR 1.56; 95 CI 1.31–1.85) or 2017 (OR 2.81; 95 CI 2.33–3.41) were more likely to use SSC than patients diagnosed in 2016. No statistically significant association was found for prior cancer diagnosis, N- and M-staging or center characteristics (see Table 2). The analysis confirmed that utilization of SSC varies between breast cancer centers, with an ICC of 0.20 after inclusion of all covariates.

**Table 2:** Results of the logistic multilevel analysis

Variable	Response option	OR	***p***-value	95 CI
Patient characteristics				
Intercept		0.82	.94	0.01–101.70
Sex	Female	Reference		
	Male	0.32	<.001	0.16–0.64
Age	Younger than 35 years	1.30	.46	0.64–2.63
	35–44 years	1.09	.63	0.77–1.54
	45–54 years	1.15	.22	0.92–1.45
	55–64 years	Reference		
	65–74 years	1.25	.05	1.00–1.56
	75–84 years	1.01	.93	0.81–1.27
	Older than 84 years	0.57	.002	0.40–0.81
Prior cancer diagnosis	Yes	0.71	.10	0.47–1.07
	No	Reference		
T-staging	TIS/DCIS	0.47	<.001	0.28–0.49
	Invasive breast cancer	Reference		
	TX	1.31	.63	0.44–3.93
N-staging	N0	Reference		
	> N0	1.07	.47	0.90–1.28
	NX	0.78	.13	0.56–1.08
M-staging	M0	Reference		
	M1	0.98	.89	0.70–1.35
Both breasts affected (metachronous and synchronous)	No	Reference		
	Yes	1.48	.03	1.03–2.11
Therapy type	Surgery with recommendation for chemotherapy	1.12	.25	0.92–1.36
	Surgery without recommendation for chemotherapy	Reference		
	No surgery	0.08	<.001	0.06–0.11
Date of diagnosis	2015	1.56	<.001	1.31–1.85
	2016	Reference		
	2017	2.81	<.001	2.33–3.41
Center characteristics				
Municipality	< 20,000 population	1.94	.53	0.25–14.96
	20,000–100,000 population	Reference		
	> 100,000 population	1.51	.56	0.38–6.10
Teaching status	None	0.26	.24	0.03–2.38
	Academic	Reference		
	University	0.28	.42	0.01–6.30
Ownership	Not-for-profit	Reference		
	Private	2.47	.50	0.18–33.22
Number of primary cases	Continuous	1.02	.12	1.00–1.04
Years since first certification	Continuous	0.91	.46	0.72–1.16
N patients	6339			
N centers	13			
ICC (nullmodel)	0.20 (0.24)			

*Note*: *OR* odds ratios, *95 CI* 95%-confidence intervals, *TIS* tumor in situ, *DCIS* ductal carcinoma in situ, *ICC* intraclass correlation coefficient

## Discussion

To our knowledge, no investigations so far have analyzed the utilization patterns of SSC in routine cancer care using individual patient data. Our analysis of utilization of SSC based on 6339 patients using quality assurance data differed considerably between centers and over time. Sociodemographic and disease-related patient characteristics were identified to explain SSC use, to some extent. These findings are in line with previous reports, based on aggregated data [11, 12].

In the multilevel model, no statistically significant differences were found for prior cancer diagnosis, nodal or metastatic staging. Literature on SSC is scarce, however, analyses with prostate cancer patients also showed no statistically significant association with nodal or metastatic-staging and utilization of SSC (Breidenbach et al., submitted) or psycho-oncological services [15]. Patients with TIS/DCIS are less likely to use SSC, compared to patients with invasive disease. This may be explained by shorter hospital stays and a lack in outpatient counseling services in the centers, resulting in less time to see a social worker. The same explanation may apply to the finding that non-surgically treated patients are less likely to utilize SSC compared to patients who received surgery. No significant differences in SSC utilization were identified between patients who received surgery with a recommendation for chemotherapy and patients who received surgery without a recommendation for chemotherapy. In an additional analysis, therapy type variables were regrouped alternatively according to surgery types (mastectomy, breast-conserving surgery, or no surgery). Again, patients without surgery were less likely to use SSC, and no significant differences were found between patients with mastectomy and breast-conserving surgery (analysis details upon request). The fact that some patients did not receive surgery might stem from the patient desire not to undergo surgery or the patients’ poor condition. Patients without surgery include a high percentage of elderly patients, which might associate with comorbidities and difficult conditions for surgery. Furthermore, findings show that patients with cancer in both breasts (metachronous and synchronous) were more likely to utilize SSC. This might be related to higher need due to more severe disease/treatment and therefore higher impairment or due to double burden when one disease follows the first. Unobserved disease characteristics, such as comorbidities, might also explain differences in SSC. Male patients are less likely to receive SSC. This is in accordance with qualitative studies suggesting shortcomings in care, particularly concerning issues of continuity of care in male patients [18], which SSC may provide with regard to rehabilitation. Patients older than 84 years were significantly less likely to utilize SSC compared to patients between 55 and 64 years old, in line with previous research showing correlations of age and the consultation of a social worker [19, 20]. However, effect sizes of the studies can barely be compared due to varying statistical methods, different health care systems in different countries and different cohorts (cited studies were published in the begin of the decade). The current findings might be related to less employment issues for elderly patients, which might indicate a lower need for support, or geriatric care already covering up support needs. Further unobserved sociodemographic factors or patients’ attitudes towards psychosocial care might also have an impact on patient’s decision of utilizing or not utilizing SSC [21, 22].

Variation of counseling frequency over time (date of diagnosis 2015, 2016 or 2017) may be interpreted as better implementation of the certification requirement in the centers and consolidation of center structures (in other words, learning effects). This is possible since, after certification requirements regarding SSC changed in 2014, low counseling frequencies were addressed in the audits and centers were asked to improve if frequencies are implausibly low. However, it should be noted that this analysis only considered a short period of time (3 years) in only 13 centers.

Further structural center characteristics were not statistically significantly associated with SSC utilization in the multilevel model whereas previous research with prostate cancer centers has pointed to associations of the teaching status and utilization of counseling services [15 and Breidenbach et al., submitted]. However, differences in utilization may also be caused by differences in the underlying processes within the centers. Abbott [23], for example, found that the location of the social worker office in the hospital plays a role regarding access of breast cancer patients to social worker services. Further explanations include differences in when and how patients are approached. There is very little data available on how such differential utilization affects later outcomes such as return to work, rehabilitation or outpatient care. An existing study using survey data, however, indicated that higher SSC frequencies in breast cancer centers were associated with decreased information needs regarding financial problems and problems with health insurance companies in multilevel analyses [10]. Future research should investigate which organizational processes within the centers might lead to higher or lower utilization rates. It should also be investigated what accounts for differences in SSC utilization of patients with other types of cancer in order to identify patterns across general oncological care or patterns specific to different oncological contexts.

It is unclear to what extent SSC is used and/or provided according to an objective need. There are no validated screening tools for SSC in use in German cancer care. Research on the effects of psychosocial counseling in routine cancer care is still at a very early stage. At the same time, issues that SSC is meant to counter have long been identified. Among these are social gradients in return-to-work and financial problems long after a cancer diagnosis [1, 24–26]. We encourage researchers and funders to collect evidence on how psychosocial care works and the impact it has on individual patients in routine cancer care, for example, by implementing screenings before and after SSC use. Further research should also investigate whether there are differences in processes (e. g. communication) within the centers that might lead to differences in SSC utilization.

The analysis of the determinants of utilization of SSC remains limited due to the lack of relevant confounders, most notably socio-economic characteristics of patients. These may be relevant particularly to the financial impact a cancer diagnosis may have on the patient [27]. However, the collection of socio-economic characteristics goes beyond quality assurance data and a study with further data collection would be necessary. Furthermore, literature on SSC is very scarce and existing literature often originates from studies in other healthcare systems or is often not differentiated from psycho-oncological care which makes it difficult to assess the current findings in relation to previous literature. The comprehensiveness of the data and the sample size are strengths of this analysis.

## Conclusion

This analysis allows a unique insight into the use of social services in the care of cancer patients and the reality of cancer care. The data collection approach applied here was used for the first time to make routinely documented data from German cancer centers available on a large scale. The multilevel findings show that there are differences in SSC utilization between breast cancer centers and that SSC utilization is associated with sociodemographic and disease-related patient characteristics.

## Supplementary Information

**Additional file 1.**

## Data Availability

The datasets generated during and/or analyzed during the current study are not available due to confidential information regarding certification processes.

## Abbreviations

95 CI: 95%-confidence interval
DCIS: Ductal carcinoma in situ
OR: Odds ratio
SSC: Social service counseling
TIS: Tumor in situ

## Footnotes

1 This law states inter alia that health care providers are obliged to ensure and further develop the quality of the services they provide. For this purpose, they are required to collect patient data.

## References

[1] Mehnert A, Brahler E, Faller H, et al. Four-week prevalence of mental disorders in patients with cancer across major tumor entities. J Clin Oncol. 2014;32:3540–6. 10.1200/jco.2014.56.0086. 10.1200/JCO.2014.56.0086 25287821

[2] Carrera PM, Kantarjian HM, Blinder VS. The financial burden and distress of patients with cancer: understanding and stepping-up action on the financial toxicity of cancer treatment. Cancer J Clin. 2018;68:153–65. 10.3322/caac.21443. 10.3322/caac.21443 PMC6652174 29338071

[3] Wright P, Downing A, Morris EJ, et al. Identifying social distress: a cross-sectional survey of social outcomes 12 to 36 months after colorectal Cancer diagnosis. J Clin Oncol. 2015;33:3423–30. 10.1200/jco.2014.60.6129. 10.1200/JCO.2014.60.6129 26282636

[4] Wright P, Wilding S, Watson E, Downing A, Selby P, Hounsome L, Wagland R, Brewster DH, Huws D, Butcher H, Mottram R, Kearney T, Allen M, Gavin A, Glaser A. Key factors associated with social distress after prostate cancer: results from the United Kingdom life after prostate Cancer diagnosis study. Cancer Epidemiol. 2019;60:201–7. 10.1016/j.canep.2019.04.006. 10.1016/j.canep.2019.04.006 31071525

[5] Mirosevic S, Thewes B, van Herpen C, Kaanders J, Merkx T, Humphris G, Baatenburg de Jong RJ, Langendijk JA, Leemans CR, Terhaard CHJ, Verdonck-de Leeuw IM, Takes R, Prins J, the NET‐QUBIC Consortium. Prevalence and clinical and psychological correlates of high fear of cancer recurrence in patients newly diagnosed with head and neck cancer. Head Neck. 2019;41:3187–200. 10.1002/hed.25812. 10.1002/hed.25812 PMC6771492 31173429

[6] Naik H, Leung B, Laskin J, McDonald M, Srikanthan A, Wu J, Bates A, Ho C. Emotional distress and psychosocial needs in patients with breast cancer in British Columbia: younger versus older adults. Breast Cancer Res Treat. 2020;179:471–7. 10.1007/s10549-019-05468-6. 10.1007/s10549-019-05468-6 31630293

[7] Cohen A, Ianovski LE, Frenkiel S, Hier M, Zeitouni A, Kost K, Mlynarek A, Richardson K, Black M, MacDonald C, Chartier G, Rosberger Z, Henry M. Barriers to psychosocial oncology service utilization in patients newly diagnosed with head and neck cancer. Psychooncology. 2018;27:2786–93. 10.1002/pon.4889. 10.1002/pon.4889 30216594

[8] Vos PJ, Visser AP, Garssen B, Duivenvoorden HJ, de Haes HCJM. Effects of delayed psychosocial interventions versus early psychosocial interventions for women with early stage breast cancer. Patient Educ Couns. 2006;60:212–9. 10.1016/j.pec.2005.01.006. 10.1016/j.pec.2005.01.006 16442463

[9] National Academies of Sciences, Engineering and Medicine (2019). Integrating social care into the delivery of health care: moving upstream to improve the Nation’s health.

[10] Kowalski C, Pfaff H, Halbach SM, Sagebiel J, Hammerschmidt P, Janßen C (2019). Sozialdienstliche Beratungsquoten und Informationsbedarfe bei BrustkrebspatientInnen – eine Mehrebenenanalyse mit PatientInnenbefragungs- und -auditdaten. Quantitative Forschung in der Sozialen Arbeit.

[11] Kowalski C, Ferencz J, Weis I, Adolph H, Wesselmann S. Social service counseling in cancer centers certified by the german cancer society. Soc Work Health Care. 2015;54:307–19. 10.1080/00981389.2014.999980. 10.1080/00981389.2014.999980 25905764

[12] Kowalski C, Ferencz J, Singer S, Weis I, Wesselmann S. Frequency of psycho-oncologic and social service counseling in cancer centers relative to center site and hospital characteristics: findings from 879 center sites in Germany, Austria, Switzerland, and Italy. Cancer. 2016;122:3538–45. 10.1002/cncr.30202. 10.1002/cncr.30202 27481151

[13] Deutsche Krebsgesellschaft, Deutsche Gesellschaft für Senologie (2020) Erhebungsbogen für Brustkrebszentren. https://www.krebsgesellschaft.de/zertdokumente.html. Accessed 01 Feb 2021.

[14] Dettmers S, Uhrig A, Voigt M, et al. Expertenstandard Psychosoziale Erstberatung onkologischer Patient*innen in der stationären Versorgung (PEOPSA). Berlin: Arbeitsgemeinschaft für Soziale Arbeit in der Onkologie (ASO), Deutsche Vereinigung für Soziale Arbeit im Gesundheitswesen e.V. (DVSG), Deutsche Krebsgesellschaft e.V.; 2018.

[15] Breidenbach C, Roth R, Ansmann L, Wesselmann S, Dieng S, Carl EG, Feick G, Oesterle A, Bach P, Beyer B, Borowitz R, Erdmann J, Kunath F, Oostdam SJ, Tsaur I, Zengerling F, Kowalski C. Use of psycho-oncological services by prostate cancer patients: a multilevel analysis. Cancer Med. 2020;9:3680–90. 10.1002/cam4.2999. 10.1002/cam4.2999 PMC7286449 32233081

[16] Kowalski C, Graeven U, von Kalle C, Lang H, Beckmann MW, Blohmer JU, Burchardt M, Ehrenfeld M, Fichtner J, Grabbe S, Hoffmann H, Iro H, Post S, Scharl A, Schlegel U, Seufferlein T, Stummer W, Ukena D, Ferencz J, Wesselmann S. Shifting cancer care towards multidisciplinarity: the cancer center certification program of the German cancer society. BMC Cancer. 2017;17:850. 10.1186/s12885-017-3824-1. 10.1186/s12885-017-3824-1 PMC5731059 29241445

[17] Lilliehorn S, Isaksson J, Salander P. What does an oncology social worker deal with in patient consultations? - an empirical study. Soc Work Health Care. 2019;58:494–508. 10.1080/00981389.2019.1587661. 10.1080/00981389.2019.1587661 30901286

[18] Halbach SM, Ernstmann N, Kowalski C, Pfaff H, Pförtner TK, Wesselmann S, Enders A. Unmet information needs and limited health literacy in newly diagnosed breast cancer patients over the course of cancer treatment. Patient Educ Couns. 2016;99:1511–8. 10.1016/j.pec.2016.06.028. 10.1016/j.pec.2016.06.028 27378079

[19] Gadalla TM. Cancer patients’ use of social work Services in Canada: prevalence, profile, and predictors of use. Health Soc Work. 2007;32:189–96. 10.1093/hsw/32.3.189. 10.1093/hsw/32.3.189 17896675

[20] Gray RE, Goel V, Fitch MI, Franssen E, Chart P, Greenberg M, Bakker D, Labrecque M, Hollowaty E, Godel R, Hampson AW. Utilization of professional supportive care services by women with breast Cancer. Breast Cancer Res Treat. 2000;64:253–8. 10.1023/A:1026548320063. 10.1023/a:1026548320063 11200775

[21] Dilworth S, Higgins I, Parker V, Kelly B, Turner J. Patient and health professional’s perceived barriers to the delivery of psychosocial care to adults with cancer: a systematic review. Psychooncology. 2014;23:601–12. 10.1002/pon.3474. 10.1002/pon.3474 24519814

[22] Bayer O, Billaudelle F, Alt J, Heß G, Specht M, Höfinghoff B, Riedel P, Wickert M, Hechtner M, Singer S. Was Männer davon abhält, ambulante Krebsberatungsstellen aufzusuchen. Eine qualitative Studie Onkol. 2020;26:1047–55. 10.1007/s00761-020-00840-4.

[23] Abbott YK Breast cancer patient access to social work. J Soc Work. 2016;17:531–43. 10.1177/1468017316649331.

[24] Heuser C, Halbach S, Kowalski C, Enders A, Pfaff H, Ernstmann N. Sociodemographic and disease-related determinants of return to work among women with breast cancer: a German longitudinal cohort study. BMC Health Serv Res. 2018;18:1000. 10.1186/s12913-018-3768-4. 10.1186/s12913-018-3768-4 PMC6311058 30594181

[25] Thong MS, Koch-Gallenkamp L, Jansen L, et al. Age-specific health-related quality of life in long-term and very long-term colorectal cancer survivors versus population controls–a population-based study. Acta Oncol. 2019;58:801–10. 10.1080/0284186X.2018.1557340. 10.1080/0284186X.2018.1557340 30736716

[26] Götz S, Dragano N, Wahrendorf M. Social inequality in reduced earning capacity among older employees: an analysis of routine data of the German statutory pension insurance. Z Gerontol Geriatr. 2019;52:62–9. 10.1007/s00391-018-01473-4. 10.1007/s00391-018-01473-4 30413945

[27] Surmann B, Lingnau R, Witte J, Walther J, Mehlis K, Winkler EC, Greiner W. Individual financial burden following a cancer diagnosis from the perspective of social services in Germany. ZEFQ. 2021:S1865921721000349. 10.1016/j.zefq.2021.02.006. 10.1016/j.zefq.2021.02.006 33820721

