# Supplementary material for: Use of social service counseling by cancer patients: an analysis of quality assurance data of 6339 breast cancer patients from 13 certified centers in Germany treated between 2015 and 2017
Source: BMC Cancer. 2021 Jun 5;21:671. doi: 10.1186/s12885-021-08396-1 (PMC8180094; doi:10.1186/s12885-021-08396-1)
Supplement: Supplementary file 1 — Additional file 1. [file 12885_2021_8396_MOESM1_ESM.docx]

**Supplementary material 1**

**Tables 1.1-1.14: Results of the univariate logistic multilevel analyses; OR = odds ratios; 95% CI = 95% confidence intervals; TIS = tumor in situ; DCIS = ductal carcinoma in situ; ICC = intraclass correlation coefficient**

**Table 1.1**

| **Variable** | **Response option** | **OR** | **p-value** | **95% CI** |
| --- | --- | --- | --- | --- |
| Intercept |  | 5.03 | <.001 | 2.87-8.83 |
| Sex | Female | Reference | | |
|  | Male | 0.37 | .004 | 0.19-0.73 |
| N patients | 6,339 | | | |
| N centers | 13 | | | |
| ICC (nullmodel) | 0.24 (0.24) | | | |

**Table 1.2**

| **Variable** | **Response option** | **OR** | **p-value** | **95% CI** |
| --- | --- | --- | --- | --- |
| Intercept |  | 5.19 | <.001 | 2.90-9.26 |
| Age | Younger than 35 years | 1.44 | .28 | 0.74-2.78 |
|  | 35-44 years | 1.15 | .40 | 0.83-1.58 |
|  | 45-54 years | 1.13 | .18 | 0.93-1.43 |
|  | 55-64 years | Reference | | |
|  | 65-74 years | 01.13 | .27 | 0.91-1.39 |
|  | 75-84 years | 0.85 | .13 | 0.70-1.05 |
|  | Older than 84 years | 0.34 | <.001 | 0.25-0.46 |
| N patients | 6,339 | | | |
| N centers | 13 | | | |
| ICC (nullmodel) | 0.24 (0.24) | | | |

**Table 1.3**

| **Variable** | **Response option** | **OR** | **p-value** | **95% CI** |
| --- | --- | --- | --- | --- |
| Intercept |  | 5.11 | <.001 | 2.91-8.96 |
| Prior cancer diagnosis | Yes | 0.55 | .002 | 0.38-1.80 |
|  | No | Reference | | |
| N patients | 6,339 | | | |
| N centers | 13 | | | |
| ICC (nullmodel) | 0.24 (0.24) | | | |

**Table 1.4**

| **Variable** | **Response option** | **OR** | **p-value** | **95% CI** |
| --- | --- | --- | --- | --- |
| Intercept |  | 5.46 | <.001 | 3.08-9.66 |
| T-staging | TIS/DCIS | 0.44 | <.001 | 0.36-0.55 |
|  | Invasive breast cancer | Reference | | |
|  | TX | 0.46 | .09 | 0.19-1.14 |
| N patients | 6,339 | | | |
| N centers | 13 | | | |
| ICC (nullmodel) | 0.25 (0.24) | | | |

**Table 1.5**

| **Variable** | **Response option** | **OR** | **p-value** | **95% CI** |
| --- | --- | --- | --- | --- |
| Intercept |  | 5.54 | <.001 | 3.12-9.82 |
| N-staging | N0 | Reference | | |
|  | > N0 | 0.90 | .18 | 0.78-1.05 |
|  | NX | 0.43 | <.001 | 0.33-0.55 |
| N patients | 6,339 | | | |
| N centers | 13 | | | |
| ICC (nullmodel) | 0.25 (0.24) | | | |

**Table 1.6**

| **Variable** | **Response option** | **OR** | **p-value** | **95% CI** |
| --- | --- | --- | --- | --- |
| Intercept |  | 5.48 | <.001 | 3.11-9.65 |
| M-staging | M0 | Reference | | |
|  | M1 | 0.31 | <.001 | 0.25-0.39 |
| N patients | 6,339 | | | |
| N centers | 13 | | | |
| ICC (nullmodel) | 0.24 (0.24) | | | |

**Table 1.7**

| **Variable** | **Response option** | **OR** | **p-value** | **95% CI** |
| --- | --- | --- | --- | --- |
| Intercept |  | 4.97 | <.001 | 2.83-8.72 |
| Both breasts affected  (metachronous and synchronous) | No | Reference | | |
|  | Yes | 1.06 | .72 | 0.77-1.47 |
| N patients | 6,339 | | | |
| N centers | 13 | | | |
| ICC (nullmodel) | 0.24 (0.24) | | | |

**Table 1.8**

| **Variable** | **Response option** | **OR** | **p-value** | **95% CI** |
| --- | --- | --- | --- | --- |
| Intercept |  | 5.57 | <.001 | 3.14-9.91 |
| Therapy type | Surgery with recommendation for chemotherapy | 1.12 | <.001 | 0.92-1.36 |
|  | Surgery without recommendation for chemotherapy | Reference | | |
|  | No surgery | 0.10 | <.001 | 0.08-0.13 |
| N patients | 6,339 | | | |
| N centers | 13 | | | |
| ICC (nullmodel) | 0.25 (0.24) | | | |

**Table 1.9**

| **Variable** | **Response option** | **OR** | **p-value** | **95% CI** |
| --- | --- | --- | --- | --- |
| Intercept |  | 3.40 | <.001 | 1.91-6-06 |
| Date of diagnosis | 2015 | 1.49 | <.001 | 1.27-1.75 |
|  | 2016 | Reference | | |
|  | 2017 | 2.41 | <.001 | 2.02-2.87 |
| N patients | 6,339 | | | |
| N centers | 13 | | | |
| ICC (nullmodel) | 0.25 (0.24) | | | |

**Table 1.10**

| **Variable** | **Response option** | **OR** | **p-value** | **95% CI** |
| --- | --- | --- | --- | --- |
| Intercept |  | 4.38 | <.001 | 2.16-8.87 |
| Municipality | < 20,000 population | 1.25 | .84 | 0.15-10.36 |
|  | 20,000-100,000 population | Reference | | |
|  | > 100,000 population | 1.44 | .56 | 0.42-4.90 |
| N patients | 6,339 | | | |
| N centers | 13 | | | |
| ICC (nullmodel) | 0.24 (0.24) | | | |

**Table 1.11**

| **Variable** | **Response option** | **OR** | **p-value** | **95% CI** |
| --- | --- | --- | --- | --- |
| Intercept |  | 5.45 | <.001 | 3.03-9.79 |
| Teaching status | None | 0.33 | .28 | 0.04-2.50 |
|  | Academic | Reference | | |
|  | University | 0.95 | .96 | 0.13-7.15 |
| N patients | 6,339 | | | |
| N centers | 13 | | | |
| ICC (nullmodel) | 0.23 (0.24) | | | |

**Table 1.12**

| **Variable** | **Response option** | **OR** | **p-value** | **95% CI** |
| --- | --- | --- | --- | --- |
| Intercept |  | 4.94 | <.001 | 2.76-8.87 |
| Ownership | Not-for-profit | Reference | | |
|  | Private | 1.10 | .93 | 0.13-9.17 |
| N patients | 6,339 | | | |
| N centers | 13 | | | |
| ICC (nullmodel) | 0.24 (0.24) | | | |

**Table 1.13**

| **Variable** | **Response option** | **OR** | **p-value** | **95% CI** |
| --- | --- | --- | --- | --- |
| Intercept |  | 2.14 | .50 | 0.23-19.52 |
| Number of primary cases | Continuous | 1.00 | .44 | 1.00-1.02 |
| N patients | 6,339 | | | |
| N centers | 13 | | | |
| ICC (nullmodel) | 0.23 (0.24) | | | |

**Table 1.14**

| **Variable** | **Response option** | **OR** | **p-value** | **95% CI** |
| --- | --- | --- | --- | --- |
| Intercept |  | 16.31 | .07 | 0.73-1.15 |
| Years since first certification | Continuous | 0.91 | .43 | 0.73-1.15 |
| N patients | 6,339 | | | |
| N centers | 13 | | | |
| ICC (nullmodel) | 0.23 (0.24) | | | |
